# Multitargeting approaches involving carbonic anhydrase inhibitors: hybrid drugs against a variety of disorders

**DOI:** 10.1080/14756366.2021.1945049

**Published:** 2021-07-30

**Authors:** Claudiu T. Supuran

**Affiliations:** NEUROFARBA Department, Sezione di Scienze Farmaceutiche, Università degli Studi di Firenze, Florence, Italy

**Keywords:** Carbonic anhydrase, inhibitors, hybrid drugs, multitargeting, anticancer agents

## Abstract

Carbonic anhydrases (CAs, EC 4.2.1.1) are enzymes involved in a multitude of diseases, and their inhibitors are in clinical use as drugs for the management of glaucoma, epilepsy, obesity, and tumours. In the last decade, multitargeting approaches have been proposed by hybridisation of CA inhibitors (CAIs) of sulphonamide, coumarin, and sulphocoumarin types with NO donors, CO donors, prostaglandin analogs, β-adrenergic blockers, non-steroidal anti-inflammatory drugs, and a variety of anticancer agents (cytotoxic drugs, kinase/telomerase inhibitors, P-gp and thioredoxin inhibitors). Many of the obtained hybrids showed enhanced efficacy compared to the parent drugs, making multitargeting an effective and innovative approach for various pharmacological applications.

## 1. Introduction

The central paradigm in drug design, at least for the last century, was the concept introduced by Paul Ehrlich of the “magic bullet”^1^. Considered the founder of chemotherapy, mainly due to his excellent work on the treatment of syphilis with arsphenamine (Salvarsan) and structurally related derivatives^2^, this scientist introduced the concept of drug target and pinpointed to the fact that an effective drug should specifically act on one target in order to manifest its therapeutic benefits. Furthermore, many of the side effects that a drug shows may be considered as being due to off-targeting, that is, interactions with diverse biomolecules than the real target, according to the magic bullet theory. As a consequence, most if not all modern drugs in clinical use were discovered considering these concepts, which continue to influence generations of medicinal chemists in the search of both new drug targets as well as compounds that should effectively, and possibly specifically interact with them for producing beneficial therapeutic effects^3^.

**Figure 1.**
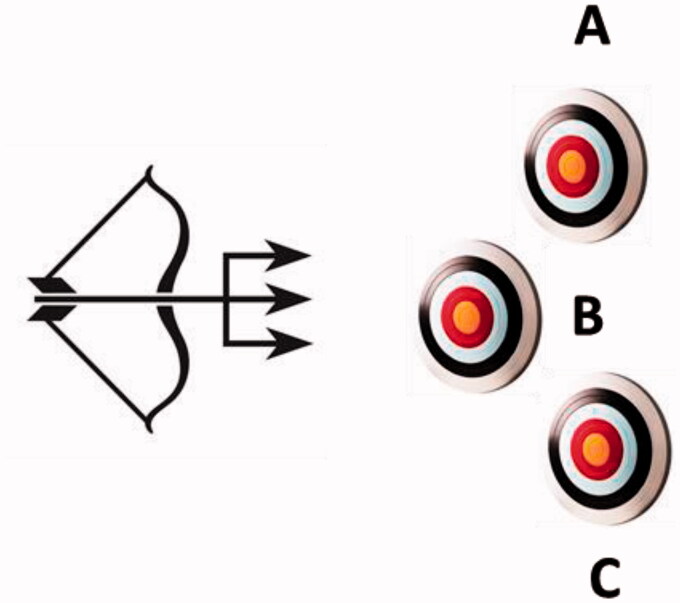
The multitargeting approach with a dual/multiple ligand, schematically shown as the triple arrow, which acts on multiple (in this case three) different targets, represented as A, B and C.

The magic bullet strategy is for sure still valid today, but started to be developed and extended in the last two decades to what is now commonly known as the multitargeting approach, which contemplates the dual/multiple (hybrid) drugs, that is, compounds that incorporate in the same molecular entity at least two different chemotypes which hit diverse drug targets^4^^,^^5^, as shown schematically in Figure 1. The advantages of such a hybrid drug may be multiple, among which the possible synergistic effect of the two (or more than two) chemotypes present in the hybrid, a more predictable pharmacokinetic profile in comparison to the administration of individual, single agents, as well as improved compliance for the patients who do not need to take many pills, etc. However, there are also challenges connected to this approach, such as for example attaining a balanced activity at each considered target, while simultaneously achieving the suitable pharmacokinetic and pharmacodynamic profiles as well as the necessary selectivity and efficacy^4^^,^^5^. For such reasons, the multiple targeting approach was initially considered mainly for multifactorial diseases such as cancer^6–8^, Alzheimer’s and other neurodegenerative diseases^9^^,^^10^, schizophrenia^11^, etc. However, more recently the approach was successfully extended to the discovery of antibiotics^12^ and antiviral drugs^13^, proving that it may be a general one.

Here I shall review the multitargeting approach of the carbonic anhydrase (CA, EC 4.2.1.1)^14^^,^^15^ inhibitors (CAIs) which have been hybridised with a variety of other diverse pharmacological agents. Indeed, the CAIs *per se* have clinical applications as diuretics^16^, antiglaucoma^17^, antiepileptic^18^, antiobesity^19^, and antitumor^20–22^ agents. Furthermore, their potential in the treatment of neuropathic pain^23^, cerebral ischaemia^24^, or as anti-infectives^25–29^ was recently highlighted, and all these aspects make them of great interest for obtaining hybrids acting on additional targets. The main reason why the multi-targeting approach is successful with CAIs is due to the fact that these enzymes are widespread in both prokaryotes and eukaryotes^14^^,^^15^, organisms in which they play crucial functions connected to pH regulation, homeostasis and metabolism, and their inhibition leads to pharmacological responses^30^. In humans, there is a multitude of isoforms (15, of which 12 are catalytically active^14^^,^^15^) specifically expressed in various tissues, and the modulation of their activities has important physiological and pharmacological applications, as already mentioned above. The field of CAs and their inhibitors was reviewed recently^31^^,^^32^ and we will not detail here these aspects, but we will directly consider the hybrid drugs based on CAIs reported so far and their various pharmacological applications.

## 2. Hybrids drugs incorporating CAIs and other pharmacophores as antiglaucoma agents

### 2.1. Antiglaucoma hybrids incorporating CAIs and NO donors

Glaucoma is a neuropathy characterised among others by elevated intraocular pressure (IOP), the only measurable clinical abnormality, which, however, if not treated, may lead to blindness or significant vision loss^17^. Avoiding the death of optical nerve neurons, responsible for the vision loss which eventually will cause blindness, is nowadays the most desirable approach for the treatment of the disease (i.e. neuroprotection) together with the lowering of IOP^17^. Systemic or topically acting sulphonamide CAIs are in clinical use for decades for the treatment of glaucoma, being among the relevant 6 classes of such drugs available nowadays^17^. In the last decades, the role of nitric oxide (NO), a radical gas involved in vasodilation and produced endogenously by the enzyme nitric oxide synthase (NOS) from arginine as substrate, has been shown to be relevant for this disease^33^. Nitric oxide acts as a potent vasodilator that impacts numerous systems throughout the body, among which also the regulation of ocular pressure. This process is similar to the systemic pressure regulation induced by this gas-transmitter in vertebrates^17^^,^^33^. In fact, the endothelium within the Schlemm’s channel (SC), involved in the outflow of the aqueous humour from the eye, has a similar shear stress sensitivity as the systemic vasculature, functioning thus as a barostat^17^^,^^33^. The NO produced by endothelial cells from SC is thus involved in regulating IOP, which, as mentioned above, is increased in the eyes of patients suffering from glaucoma^17^. Thus, the idea to combine CAIs, clinically used antiglaucoma agents, as mentioned above, with NO-donating compounds, a relatively underexplored class of ocular drugs till recently, led to the development of hybrid drugs which indeed showed excellent IOP and neuroprotective action^34^. The increased vasodilation, coupled with potential anti-inflammatory and anti-platelet effects of NO itself (and probably also of the sulphonamides^35^) are only some of the various activities that a hybrid incorporating a NO-donor moiety and a CAI can offer^36–39^.

**Figure 2.**
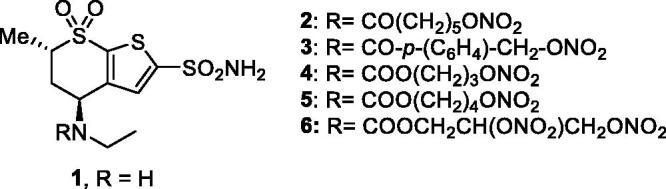
Structure of the nitrate ester derivatives of dorzolamide **1** (R = H) of types **2–6**.

**Figure 3.**
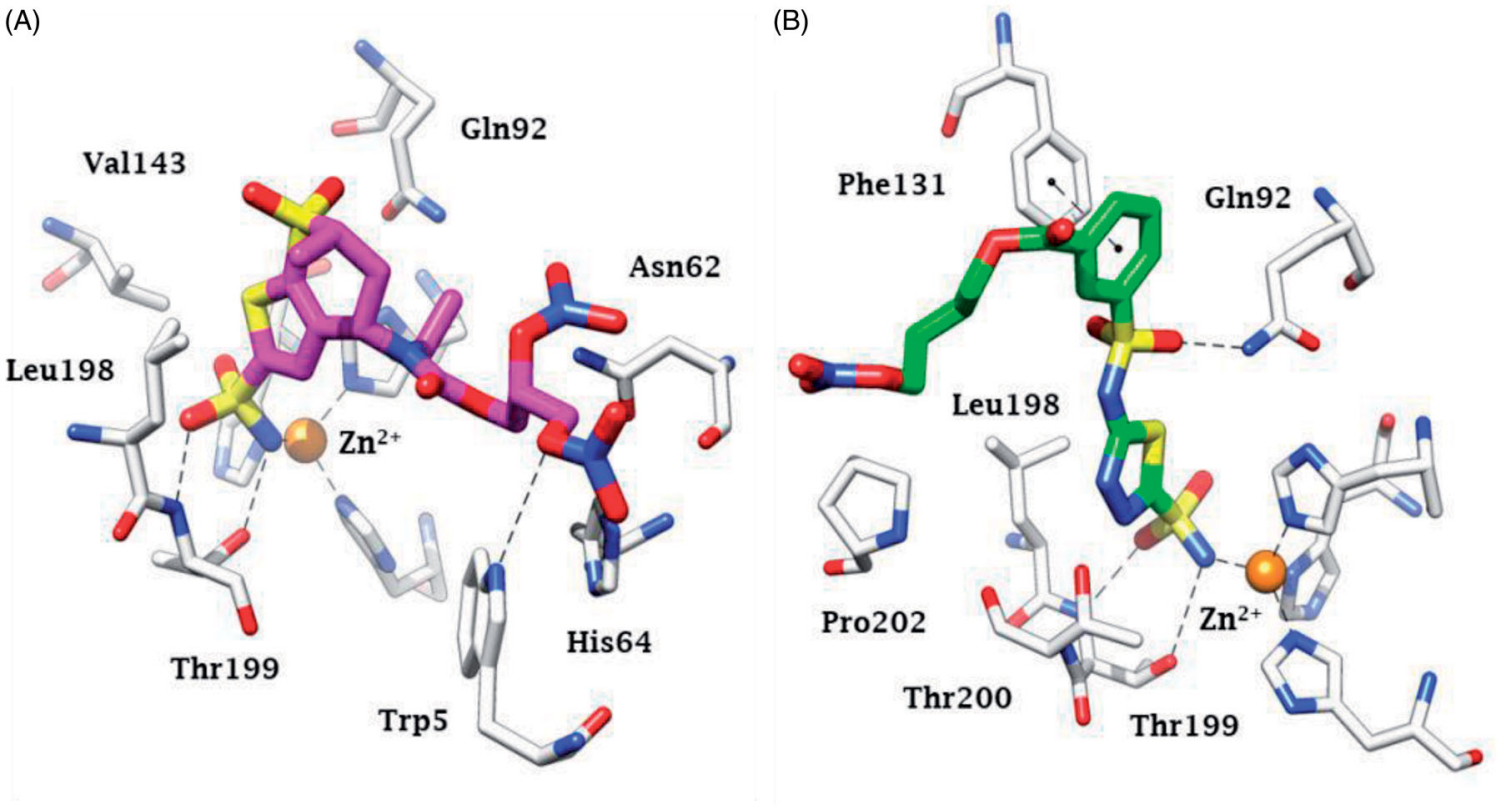
Binding of sulphonamide–NO donors to hCA II as determined by X-ray crystallographic experiments. (A) Compound **6** (PDB 3K2F) in magenta, and (B) sulphonamide **8** (PDB 3NI5) is shown in green. The zinc ion from the CA active site is shown as a gold sphere, being coordinated by three His residues (His 94, 96 and 119 – not numbered in the figure for the sake of simplicity) and the fourth metal ion ligand being the deprotonated sulphonamide from the inhibitor. The other amino acid residues involved in contacts with the inhibitors are also highlighted.

The first compounds to incorporate these two diverse chemotypes were based on the dorzolamide **1** saffold^36^. Dorzolamide **1** itself is a sulphonamide CAI (Figure 2) in clinical use since 1995 as eye drops for the topical treatment of glaucoma^16^^,^^17^. Hybridisation of **1** by means of various NO-donating moieties incorporated in nitrate ester functionalities, as the compounds **2–6** (Figure 2), led to hybrids with effective, low nanomolar CA inhibitory properties against the isoforms involved in aqueous humour production, such as CA II and IV, and also showed effective IOP-lowering properties in an animal model of glaucoma, that is, normotensive rabbits^36^. Furthermore, the X-ray crystal structure of some of these sulphonamide–NO donor hybrids, bound to hCA II, were resolved and are shown in Figure 3^36,37^.

**Figure 4.**
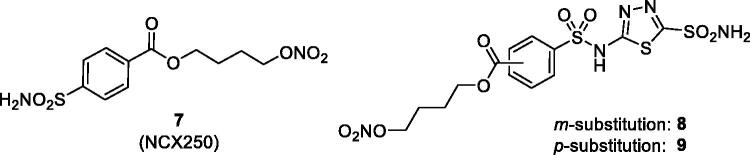
Chemical structure of aromatic/heterocyclic sulphonamides incorporating NO-donating moieties of the aliphatic nitrate ester type, **7–9**.

In subsequent studies, various other CAI–NO donating compounds were synthesised and investigated as CAIs and IOP lowering agents, among which 4-carboxy-benzensulphonamide, 1,3,4-thiadiazole-2-sulphonamide, 4–(2-carboxyethyl)-benzensulphonamide and 4-hydroxybenzensulphonamides (Figure 4)^37^^,^^38^. Some of these derivatives, such as compounds **7–9** were low nanomolar *in vitro* CA inhibitors of isoforms involved in aqueous humour formation, such as CA II, CA IV and CA XII^37^^,^^38^. By means of X-ray crystallographic studies, the factors associated with their significant inhibitory activity were also revealed, as shown in Figure 3(B) for the adduct of **8** with hCA II (h = human isoform)^37^.

In fact, for both hybrids incorporating sulphonamides and NO donating moieties which have been characterised by crystallography when bound to hCA II (compounds **6** and **8**, see Figure 3), the deprotonated sulphonamide group of the hybrid was observed bound to the zinc ion, the organic scaffold was participating in a large number of favourable interactions with amino acid residues from the active site (shown in detail in Figure 3), and the tail NO-donating fragment of the hybrid was also observed intact, demonstrating that the hybrids were stable enough for exerting their dual effect^36^^,^^37^.

Compound **7**, also known as NCX-250^38^, was investigated in detail as an antiglaucoma agent from the pharmacological viewpoint. In a rabbit model of transient ocular hypertension NCX-250 was 2 times a more effective IOP lowering agent compared to dorzolamide, a clinically used drug, as mentioned above^17^^,^^38^. It also reduced short-term elevated IOP in another animal model of the disease, that is, the rabbit with carbomer-induced glaucoma^38^. The NO-donating properties of the compound were also assessed using isosorbide mononitrate as standard NO-donor, being observed a slow but steadfast release of the gaso transmitter. It was thus concluded that the effective IOP lowering caused by NCX-250 treatment is due both to its CA inhibitory effects and to the vasodilation induced by the liberated NO, as expected for this type of hybrid drug^38^. In the same study was then reported that the chronic administration of NCX-250 as 2% eye drops in carbomer-induced glaucomatous New Zealand albino rabbits led to a significant and durable IOP lowering, which was superior to that observed with the single, standard drugs, dorzolamide or isosorbide mononitrate^38^. By using echo-doppler measurements of the ophthalmic artery in animals treated with NCX-250, reduced systolic and diastolic velocities were observed, suggesting a beneficial effect of this class of hybrids incorporating sulphonamide CAIs and NO-donating nitrate esters for supplying blood to the optic nerve, an effect which has not been observed when dorzolamide alone was administered to the experimental animals.

**Figure 5.**
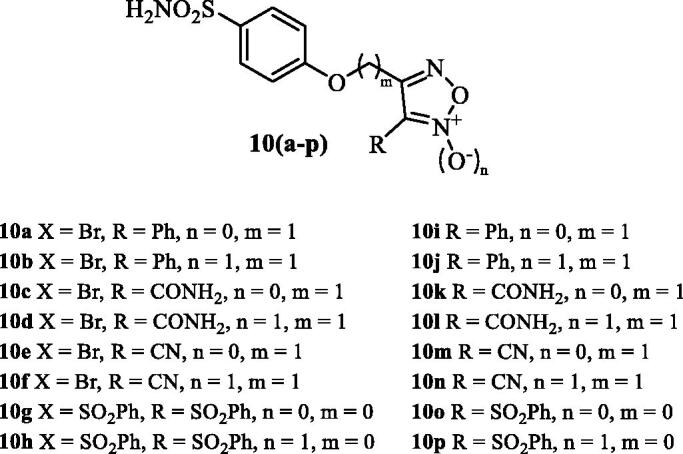
Sulphonamides **10 (a–p)** incorporating furazan and furoxan as NO-donating moieties.

Another study reported thereafter sulphonamide CAIs incorporating furazan and furoxan derivatives as NO-donating moieties, of types **10 (a–p)** (Figure 5)^39^. Although effective CA inhibitory effects (in the low nanomolar range) and IOP lowering (slightly more effective than dorzolamide) were observed with hybrids of type **10**^39^, they were not superior to the nitrate esters mentioned above, which are easier to be prepared and more stable compared to the furazans^38^^,^^39^.

### 2.2. Sulphonamides conjugated with prostaglandin F receptor agonists

**Figure 6.**
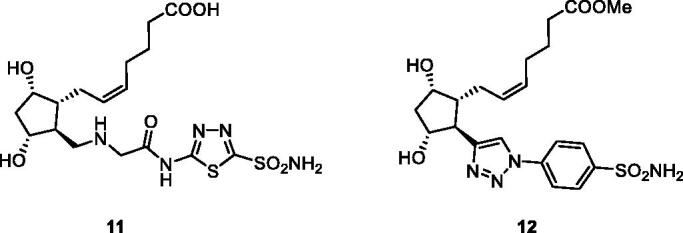
Prostaglandin receptor agonists–sulphonamide CAIs hybrids of types **11** and **12**.

The prostaglandin F (PGF) receptor agonists (PGFR) constitute a well-known class of antiglaucoma drugs, with several compounds in clinical use for more than two decades, the best known one being latanoprost^40^. They decrease IOP by increasing the uveoscleral ocular aqueous outflow^17^. Long et al.^41^ proposed a hybridisation of sulphonamide CAIs with PGFR agonists as a new approach for the treatment of glaucoma. The heterocyclic (1,3,4-thiadiazole-sulphonamide) and aromatic (benezenesulphonamide) hybrids of types **11** and **12**, Figure 6, have been obtained, which possessed good ocular permeability, but no *in vivo* antiglaucoma data and detailed CA inhibition experiments have been reported to date for these presumably dual-acting compounds^41^. However, this strategy represents and interesting and innovative approach for the management of glaucoma.

### 2.3. CAI–β-adrenergic receptors antagonists hybrids

**Figure 7.**
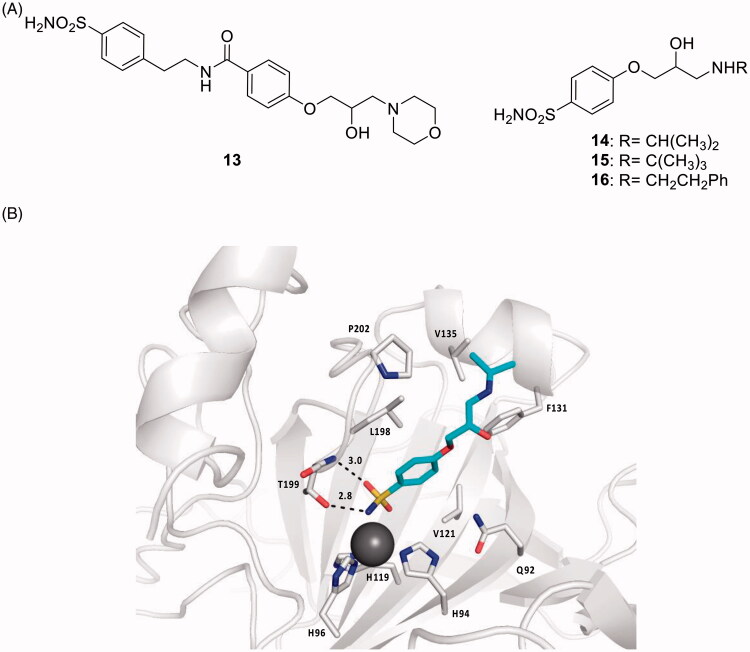
(A) Sulphonamide CAI–β-blocker hybrids of types **13–16**. (B) X-ray crystal structure for the adduct of **14** with hCA II: the zinc ion (gray sphere) with its three protein ligands (His94, 96, 119) are evidenced. The inhibitor **14** (blue sky) is coordinated with the deprotonated sulphonamide moiety to the metal ion, and its scaffold participates in a range of favourable interactions with active site residues, such as Thr199, Leu198, Phe131, Val135. Hydrogen bonds are shown as dashed lines.

One of the mostly used antiglaucoma drug eye drops formulations is COSOPT^®^, which combines two drugs, the β-adrenergic receptor (AR) antagonist timolol and the CAI dorzolamide^17^. β-Blockers such as timolol and many other derivatives structurally related to it reduce IOP through the blockade of sympathetic nerve endings within the ciliary epithelium, whereas the CAIs reduce the secretion of the aqueous humour due to the inhibition of the ciliary process CA isoforms CA II, IV and XII involved in the production of bicarbonate present in the aqueous humour^17^. Thus, a multi-targeted drug design strategy of hybrid drugs incorporating CAIs of the sulphonamide type and β-ARs antagonists was proposed by Nocentini et al. (Figure 7(A))^42^. Dual derivatives of types **13–16**, showed a remarkable CA inhibitory potency, in the nanomolar range, against isoforms hCA II and XII (involved in glaucoma), also possessing an acceptable affinity to β-ARs (of type β1 and β2) in the low micromolar range, comparable to that of the racemic, clinically used β-blocker drug atenolol^17^^,^^42^. The X-ray crystal structure of hCA II in complex with the dual compound **14** was determined and explained why the compound is a highly effective CAI against hCA II (Figure 7(B))^42^. These derivatives showed an IOP lowering effect comparable or better than those of dorzolamide, timolol and their combination (COSOPT), 60 min post-treatment. On the other hand, for some of these dual-acting compounds, the IOP lowering efficacy was two-fold higher compared to the parent molecules after 1 and 2 h post-administration, demonstrating thus the great efficacy and longer duration of action of the multi-targeted strategy with this type of hybrids^42^.

## 3. Hybrids of CAIs with anti-inflammatory agents

### 3.1. CAI–CO releasing molecules (CORMs) hybrids

Similar to NO discussed in the preceding paragraph, CO is another gaso-transmitter involved in a multitude of physiological processes, although being a highly toxic molecule^43^. CO, unlike NO, is a rather stable gas and it is endogenously produced through the degradation haem under the action of the enzyme haem oxygenases (HO; EC 1.14.99.3), more precisely its isoforms HO1 and HO2^44^. These enzymes and the CO produced through their activity, play a protective role against intracellular oxidative stress, and an overexpression of HO1 was reported in several inflammatory and autoimmune diseases^45^. Thus, although CO is highly poisonous compound due to its strong affinity for haemoglobin (Hb), this gas also binds to other haem-containing proteins, such as the soluble guanylyl cyclase (sGC), the NO synthase (NOS), the NADPH oxidase as well as mitochondrial cytochrome C oxidase, and this probably endows CO with cytoprotective and homeostatic antiiflammatory properties^46^. Indeed, in the last decades, CO releasing molecules (CORMs) which allow a controlled release of small amounts of this toxic compound started to be considered for therapeutic applications^46^^,^^47^. The alkyne hexacarbonyl dicobalt(II) complexes are the most well-known CORMs, although many other such chemotypes have been developed^46^^,^^47^.

**Figure 8.**
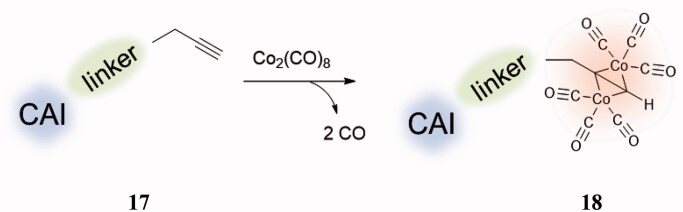
General strategy for obtaining CAI–CORM hybrids **18** based on the hexacarbonyl dicobalt(II) complexes.

Thus, a strategy to obtain CAI–CORM hybrids, which may exploit the pharmacological properties of both chemotypes, was recently proposed by Berrino et al.^48^ and it is shown schematically in Figure 8. The idea is based on the fact that the dicobalt(II)octacarbonyl ha a high affinity to form complexes with terminal alkynes, such as **17** (losing 2 CO molecules) leading thus to derivatives of type **18** (Figure 8), which are stable but may release CO depending on the substitution pattern at the alkyne moiety^46–48^.

**Figure 9.**
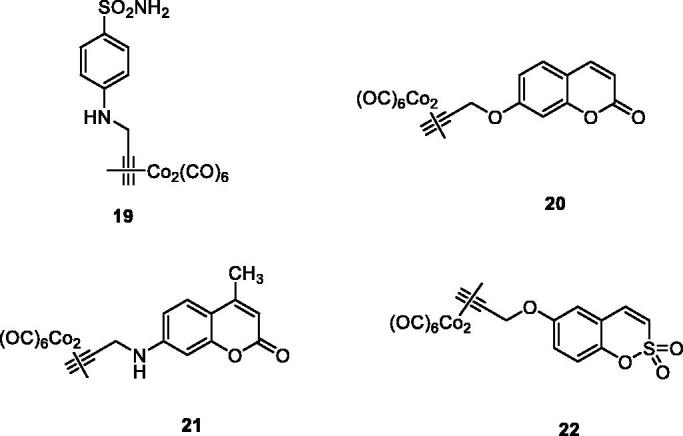
CAI–CORM hybrids of types **19–22** incorporating sulphonamide, 7-hydroxycoumarin, 7-aminocoumarin and 6-hydroxysulphocoumarin as scaffolds with CA inhibitory properties and hexacarbonyl dicobalt(II) as CO releasing fragment.

All common CAIs, such as the aromatic sulphonamides (compound **19**), hydroxycoumarins (compound **20**), 7-aminocoumarins (derivative **21**) and 6-hydroxysulphocoumarins (derivative **22**) have been hybridised in this way with hexacarbonyl dicobalt(II) moieties, as shown in Figure 9. The isoforms hCA I, II, IV, IX, XII were effectively and sometimes selectively inhibited by such derivatives, with efficacies in the nanomolar range, whereas the pain-relieving properties of several hybrids were also assessed in an animal model of rheumatoid arthritis (RA), by using the paw-pressure and incapacitance tests, which demonstrated an efficacy similar or slightly better than that of the clinically used drug ibuprofen, belonging to the cyclooxygenase (COX) inhibitors^48^. In subsequent papers from the same group^49^^,^^50^, it has been demonstrated that the CAI–CORM hybrids modulate lipopolysaccharide (LPS) stimulation in macrophages, exerting beneficial anti-inflammatory effects and counteracting inflammatory stimuli by reducing the release of tumour necrosis factor alpha (TNF-α), a pro-inflammatory protein^49^. Tendon recovery after injury is another inflammation model in which the utility of the CAI–CORM compounds was recently demonstrated^50^. Tenocyes (tendon cells) were shown to be protected by CAI–CORM hybrids from oxidative stress induced by inflammation and to have an enhanced viability after treatment with the hybrid drug, which may be another useful application of these compounds.

### 3.2. CAI–cyclooxygenase inhibitor (non steroidal anti-inflammatory drugs, NSAIDs) hybrids

**Figure 10.**
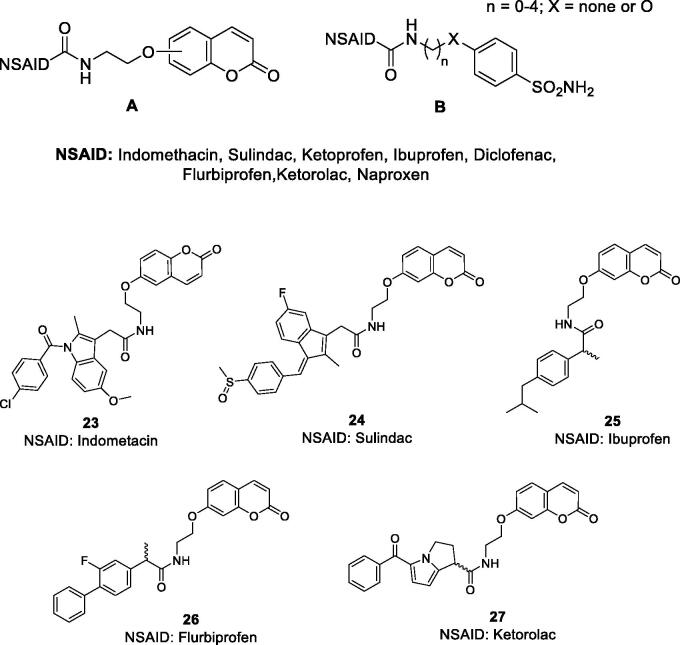
General structures of the COX inhibitor-CAI hybrids **A** and **B**, and several coumarin–NSAIDs hybrids of type **23–27**.

Cyclooxygenases (COXs) are involved in the biosynthesis of prostaglandins, essential inflammation modulators, and their inhibition by COX inhibitors, also known as non steroidal anti-inflammatory drugs (NSAIDs) is one of the main therapeutic approach for controlling various inflammatory processes, including pain, fever, arthritis, etc.^51–56^. Several CA isoforms, among which CA II, IV, IX and XII were demonstrated to be involved in arthritis-like diseases^57^, being overexpressed in the synovial fluid and synoviocytes from patients suffering of rheumatoid arthritis (RA). Thus, hybrids were designed to target such CA isoforms involved in this condition, incorporating both a CA-binding moiety of the coumarins or sulphonamide type, and a COX inhibitor of the NSAID type such as the carboxylic acid derivatives indomethacin, sulindac, ibuprofen, flurbiprofen, ketoprofen, diclofenac, ketorolac, naproxen, etc. (Figure 10)^51–56^.

Both 6- and 7-hydroxy-coumarins, such as those shown in Figure 10^51,53^ as well as the corresponding derivatives incorporating esters instead of amides^52^, but also the sulphonamide–COX inhibitor hybrids (of type **B** in Figure 10)^54^ were obtained and investigated for their stability in plasma and for inhibition of the CA isoforms involved in inflammatory processes (hCA II, IV, IX and XII), and in some cases also for the inhibition of COX1/2 isoforms^56^^,^^57^. The hybrids were shown to be quite stable to hydrolysis (except for some coumarin esters) and to potently inhibit both CAs and COX isoforms involved in inflammation, with high efficacy being thus rather equilibrated hybrids. But more importantly, in several inflammation models, they showed excellent *in vivo* activity, superior to the parent compounds (CAI alone, COX inhibitor alone and the combination of the two drugs), proving the efficacy of the multitargeting approach^51–56^. Indeed, some of the obtained coumarin and sulphonamide hybrids, were assessed by means of the paw-pressure and incapacitance tests in a rat *in vivo* model of RA^47^. Potent antihyperalgesic effects were observed for all of them, in a dose-dependent manner, starting already at very low concentrations of hybrids, as low as 1 mg kg^−1^. For example, the 7-coumarin substituted ibuprofen derivative **25** showed long-lasting antihyperalgesic effects, in the two *in vivo* models mentioned above, superior to both ibuprofen or the 7-hydroxycoumarin (and their combination)^51^^,^^55^. Some of these derivatives as well as the corresponding sulphonamide hybrids also showed potent anti-inflammatory activity in a lung fibrosis animal model^56^, making this type of hybrids of great interest as novel anti-inflammatory agents.

## 4. Antitumor hybrid drugs incorporating CAIs conjugated with other cheotypes

### 4.1. CAI–cytotoxic agents hybrids

At least two CA isoforms, hCA IX and XII, are overexpressed in solid tumours as a consequence of the hypoxic environment and activation of the hypoxia inducible factor (HIF) signalling cascade^20–22^. These two tumour-associated CAs were validated as antitumor/antimetastatic drug targets, with at least one small molecule compound, the sulphonamide SLC-0111 in Phase Ib/II clinical trials for the management of hypoxic, metastatic solid tumours^58^. Since cancer is a multifactorial disease, and as CA IX/XII were only recently validated as drug targets, a multitude of hybridisation approaches involving CA IX/XII inhibitors and other antitumor chemotypes were proposed in the last period.

**Figure 11.**
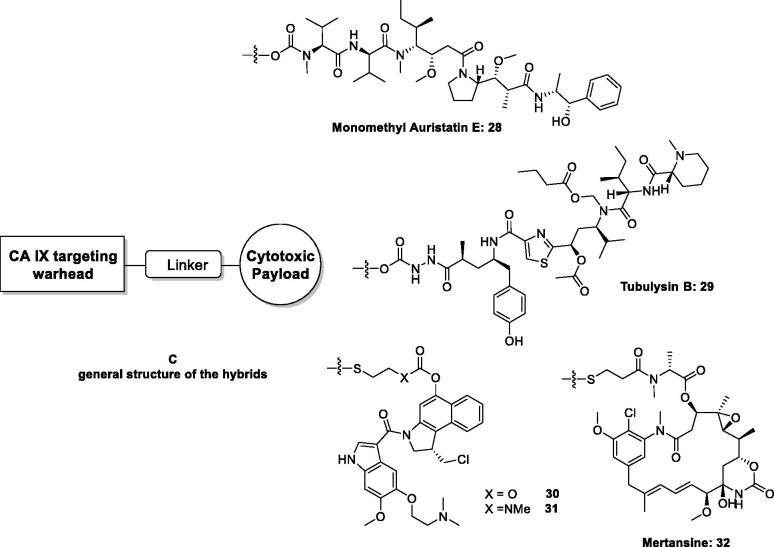
General strategy for hybridisation of CAIs with cytotoxins in compounds of type **C**. CA IX/XII–cytotoxic agent hybrids, incorporate toxic payloads **28–32,** whereas the CA inhibitory warheads may be benzenesulphonamide or 1,3,4-thiadiazole-2-sulphonamide derivatives (although coumarins and other CAI chemotypes can be used). The linker is usually a tetrapeptide incorporating water-solubilizing residues (Asp, Arg, Cys).

The high affinity of aromatic/heterocyclic sulphonamides acting as CAIs for CA IX and XII, which are found in rather low concentration in most normal tissues but are overexpressed in many hypoxic tumours^20–22^ led Neri’s and other groups to propose them for hybridisation with cytotoxic payloads possessing a variety of chemical functionalities in order to specifically target tumours without affecting the healthy tissues Figure 11^59–63^. The cytotoxins included in these studies were monomethyl auristatin (compound **28** in Figure 11)^60^^,^^61^, tubulysin B (scaffold **29** in Figure 11)^62^, the maytansinoids **30** and **31**^59^, as well as mertansine **32**^59^ (Figure 11). All these toxins are highly effective in killing tumour cells but they are equally toxic to normal cells, and for this reason are impossible to be used in therapy. However, the conjugation of these toxins with CAIs of the sulphonamide type as “warheads,” through oligopeptide linkers as shown in Figure 11, led to dual agents which showed effective anticancer effects due to the action of the delivered cytotoxin only within tumour cells, as well as due to the inhibition CA IX and XII, which, as mentioned above, are tumour-associated and are present in low amounts in normal tissues, thus interfering with the metabolism and pH regulation in tumours^20–22^^,^^58–63^.

Hybrids as those shown in Figure 11, incorporating aromatic/heterocyclic sulphonamides as CA inhibitory chemotype, possessed low nanomolar affinity for CA IX as well as *in vivo* activity, in SK-RC-52 renal cell carcinoma cancer xenograft models of cancer in experimental animals^59–61^^,^^63^.

### 4.2. Dihydroartemisinin–CAI hybrids

**Figure 12.**
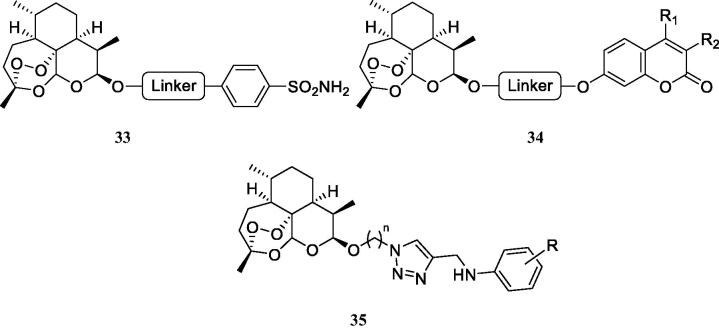
DHA–CAI hybrids incorporating sulphonamide (**33** and **35**) and coumarin moieties (**34**) as CA inhibitory fragment. In compounds 35R = sulfamoyl, in *meta* or *para* positions. The linkers may be 1,2,3-triazole based moieties obtained by click chemistry, –O–(CH_2_)*_n_*–O–, *n* = 2–4, etc.

Artemisinin and dihydroartemisinin (DHA), which is more hydrophobic than the parent compound, are well-known antimalarial agents, which have been also shown to possess cytotoxic activity against several cancer cell lines^64^. Guo’s group reported interesting hybrids incorporating DHA and CAIs of the type shown in Figure 12^64–66^.

The sulphonamides of type **33** and **35** have been tested as hCA I, II, IX and XII inhibitors and showed generally low micromolar inhibitory action, and anti-proliferative activity against several cancer cell lines, such as MDA-MD-231 (breast), HT-29 (colon) and no toxicity against the normal MCF-10A epithelial cells^64^. The coumarins **34** were not tested as CAIs, although these compounds are known to possess remarkable such activity^14^^,^^15^^,^^67^^,^^68^, coupled with selectivity for the inhibition of the tumour-associated versus the cytosolic isoforms. Thus, the coumarins were only tested for their antiproliferative activity against the same cancer cell lines mentioned for **35**, showing interesting activities^65^^,^^66^. Some of the coumarins reported in these interesting studies were docked within the CA IX active site as hydrolysed derivatives, as indeed, the inhibition mechanism by this class of compounds involves their hydrolysis at the lactone ring and occlusion of the CA active site entrance by the obtained hydroxycinnamic acids^67^^,^^68^. However, the docking performed in the mentioned studies^65^^,^^66^ reported the carboxylate moieties of the hydrolysed coumarin as coordinating the active site zinc ion, which is not in agreement with the X-ray crystallographic data mentioned above^67^^,^^68^.

### 4.3. Other CAI–anticancer drug hybridisation approaches

Some of the most widely used anticancer drugs nowadays are the tyrosine kinase inhibitors and the tubulin polymerisation inhibitors, with many new representatives of these classes being launched each year^69^. Thus, in this paragraph I will examine a set of hybridisation approaches which involve CAIs and several other, rather heterogeneous classes of antitumor agents, among which the epidermal growth factor receptor (EGFR) antagonists (this protein has kinase activity)^70^, the 15-lipoxygenase (15-LOX)/COX-2 (multitargeting of tree different proteins) inhibitors^71^, the telomerase inhibitors^72^, the P-glycoprotein (P-gp) inhibitors^73^, and the thioredoxin inhibitors^74^^,^^75^.

**Figure 13.**
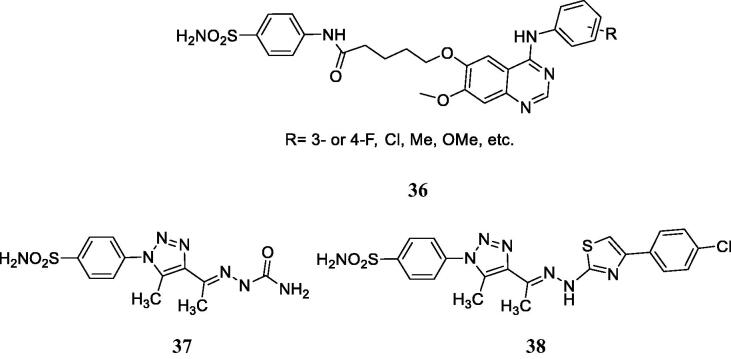
Multitargeting compounds incorporating the kinase inhibitory fragment from erlotinib and sulphonamide CAIs of type **36** and compounds **37** and **38** targeting CAs, COX-2, and 15-LOX.

Zhang et al.^70^ reported hybrids incorporating the tyrosine kinase inhibitory fragments found in erlotinib and gefitinib, clinically used anticancer agents^69^ and benzenesulphonamides with CA inhibitory activity, of types **36** (Figure 13) which were shown to possess antiproliferative activity against several cell lines, such as A549, A431 and H1975. Few of the compounds were also tested for the inhibition of EGFR (wild type and mutant) and hCA II and IX, showing nanomolar activity for the first protein, and micromolar ones for the CAs. However, the docking of some of the primary sulphonamides within the hCA IX active site was erroneously performed by these authors^70^. The sulphonamide was docked as neutral molecule and not as anion (although sulphonamides bind to these enzymes in the anionic, sulphonamidate form^14–16^) and as a consequence they were reported to inhibit CAs due to the hydrogen bonds formed with Thr199, Thr200 and several other amino acid residues, which is completely erroneous.

Elzahhar et al.^71^ reported a multitargeting strategy involving three different targets: CAs, COX-2 and 15-LOX, all enzymes found to be overexpressed in some types of tumours. Some of the reported derivatives, such as **37** and **38** (Figure 13) showed nanomolar CA IX/XII and COX-2 inhibitory action and micromolar inhibition of 15-LOX, and possessed effective antiproliferative action against hepatic (HepG2), breast (MCF7) and lung (A549) cancer cell lines^71^. These compounds were also docked within the active sites of the three enzymes, showing favourable interaction, but as for the preceding study, the docking with the CA IX active site was performed with the neutral and not the deprotonated sulphonamide. However, the interaction with the zinc ion was in this case observed, being in fact one of the main factors responsible for the effective inhibition of these enzymes^71^.

**Figure 14.**
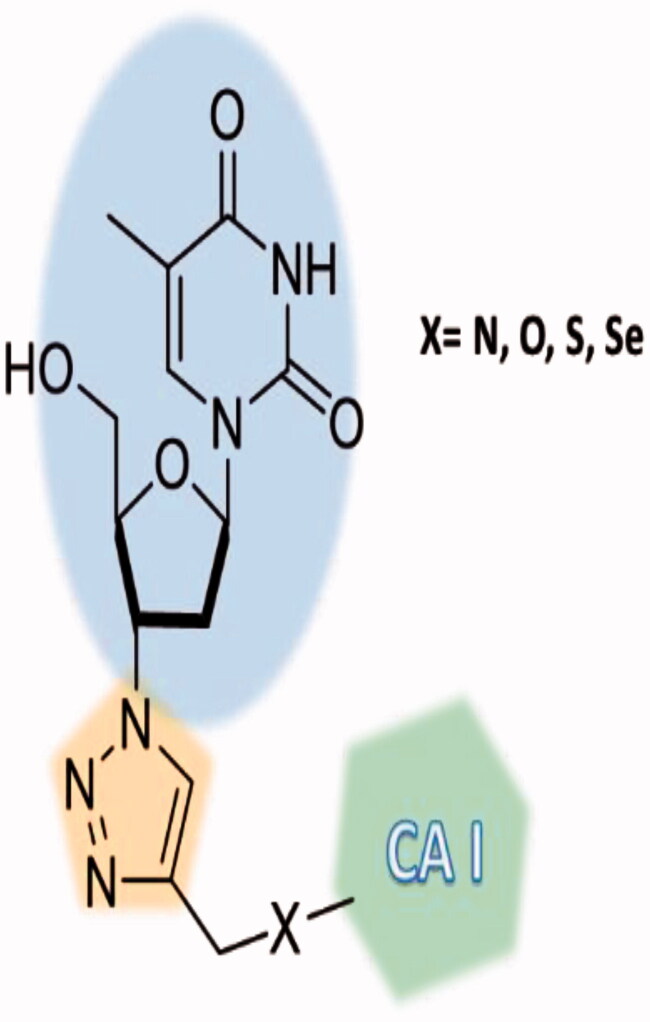
Hybridisation of CAIs and telomerase inhibitors of the AZT type.

**Figure 15.**
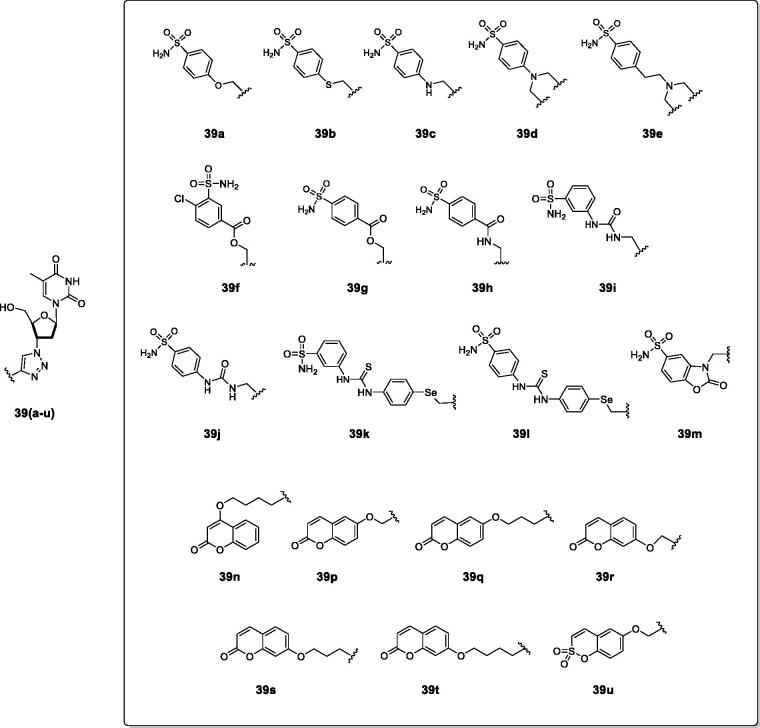
Telomerase-CAI hybrids **39 (a–u)** incorporating sulphonamide, coumarin, sulphocoumarin fragments with CA inhibitory action, AZT as telomerase inhibitor and various linkers (ether, thioether, amine, amide, ester, selenoether, carbamate, etc.).

Berrino et al.^72^ reported a highly innovative hybridisation approach of CAIs with telomerase inhibitors based on the azidothimidine (AZT) scaffold (Figures 14 and 15) by using click chemistry procedures.

**Figure 16.**
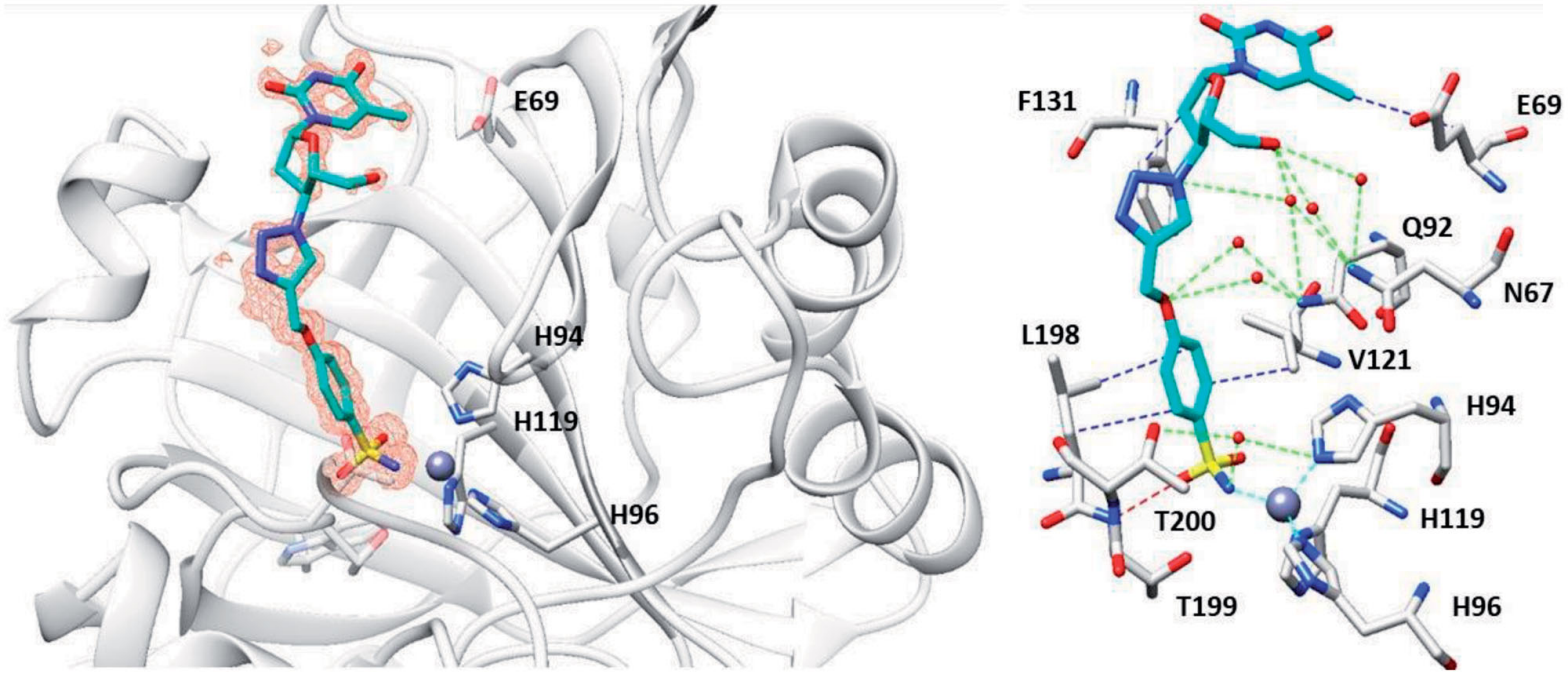
The adduct of hCA II with **39a** as determined by X-ray crystallography. Left: hCA II active site, with the zinc ion (gray sphere) and its histidine ligands (His94, 96 and 119) and the inhibitor showed with its electronic density. Right: detailed interactions in which the sulphonamide **39a** participates with amino acid residues from the enzyme active site. Red spheres are water molecules, dashed lines represent hydrogen bonds. Residues involved in the binding are evidenced.

As seen from Figure 15, a large number of telomerase-CAI hybrids of type **39 (a–u)** were reported, which incorporate the main CA inhibitory chemotypes, of the primary sulphonamide, coumarin, and sulphocoumarin type^72^. The telomerase inhibitor was on the other hand always based on the AZT fragment, but the linkers between the two chemotypes were highly variable, as shown in Figure 15. Many such derivatives showed low nanomolar CA IX/XII inhibition and potent antitelomerase activity in PC-3 and HT-29 cell lines, with IC_50_ values ranging from 5.2 to 9.1 μM against this target^72^. Some of the sulphonamides were also crystallised in complex with some CA isoforms, such as hCA II, demonstrating the structural reasons of their strong affinity for this target^72^, as shown in Figure 16.

**Figure 17.**
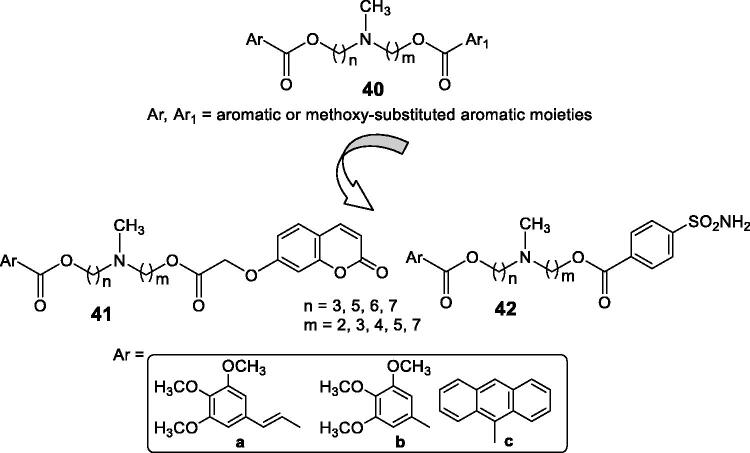
CAI–P-gp inhibitor hybrids of the coumarin **41** and sulphonamide **42** types.

Teodori et al.^73^ proposed hybrids incorporating P-gp inhibitors of the amine (**40**) type and CAIs of the coumarin (**41**) and sulphonamide (**42**) types, showed in Figure 17.

The P-glycoprotein (P-gp) is involved in the resistance problems observed with many antitumor agents, as this transporter extrudes the anticancer drugs from the cells^73^. Tertiary amines of the verapamil type (general structure **40**) act as effective inhibitors of P-gp, and this was the rationale for obtaining hybrids of P-gp inhibitors and CAIs targeting the tumour-associated enzymes CA IX and XII. Indeed, compounds of types **41** and **42** incorporate the *N,N*-bis(alkanol)amine diester scaffold to which the CA inhibitory fragment has been appended. They showed effective CA IX/XII inhibitory action, some of them in the low nanomolar range, as well as inhibitory activity against P-gp. In a rhodamine-based test for evaluating multidrug resistance to antitumor agents in LoVo/DOX cells, that overexpress both P-gp and CA XII, some of the coumarin derivatives **41** were able to revert the phenomenon, leading to an increased uptake of doxorubicin within the tumour cell, demonstrating thus the efficacy of the hybridisation approach^73^.

**Figure 18.**
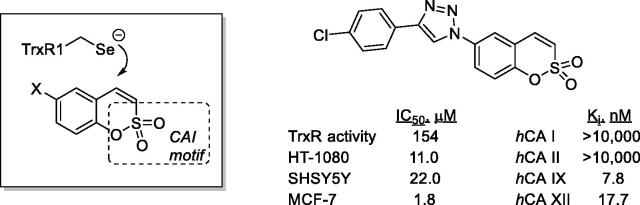
Sulphocoumarins targeting tumour-associated CAs and thioredoxin.

Recently, Krasavin et al.^74^^,^^75^ proposed another interesting multitargeting approach of two enzymes involved in cancerogenesis: thioredoxin (TrxR, which is overexpressed in cancers as a defence against oxidative stress) and CA IX/XII, involved in metabolism and pH regulation in tumours (Figure 18). Indeed, variously substituted sulphocoumarins acted as low nanomolar hCA IX/XII inhibitors and showed micromolar activity against TrxR, a selenium-containing enzyme (Figure 18). The effiocacy of the dual targeting was also tested *ex vivo*, in the pancreatic cancer cell line PANC-1, showing superior efficacy to the single agents^75^.

## 5. Conclusions

Due to the fact that the CAs are enzymes involved in a variety of physiologic and pathologic processes, and since highly effective CAI inhibitors are available belonging to many chemical classes^76–78^, their hybridisation with other pharmacologic agents, in a multitargeting approach, saw important developments over the last 15 years. The most advanced applications are those in the field of glaucoma, since the sulphonamide CAIs were combined with NO-donating moieties, PG receptor agonists, and β-blockers. Many of the obtained hybrids showed excellent affinity for both targets and an enhanced biological activity, in this case IOP lowering, in animal models of glaucoma. However, there are still several classes of antiglaucoma agents which have not yet been hybridised with CAIs, such as for example the α-adrenergic agonists, the rock-kinase inhibitors, etc., and they should offer interesting possibilities to obtain novel antiglaucoma drugs. Interesting developments were also registered for the hybrids of CAIs with anti-inflammatory agents such as CORMs, for obtaining compounds which release in a controlled and slow manner CO, as well as with classical NSAIDs belonging to the aryl-acetic or aryl-propionic acid drugs. Sulphonamides, coumarins and sulphocoumarin CAIs were hybridised with such derivatives by using different derivatization techniques, which showed increased anti-inflammatory action in various disease models in experimental animals.

The anticancer field also saw interesting multitargeting approaches, as sulphonamide/coumarin CAIs have been hybridised with cytotoxic agents, kinase inhibitors, telomerase inhibitors, P-gp inhibitors or thioredoxin inhibitors. Many such derivatives showed enhanced anti-proliferative activity compared to the single individual agents or their combination, proving the efficacy of the multitargeting. However, there are many other classes of pharmacological agents which would be amenable to obtaining hybrids with CAIs which were not yet investigated, and presumably in the future some of them will be explored.
